# Bilateral correction of asphyxiating thoracic dystrophy

**DOI:** 10.1093/jscr/rjac352

**Published:** 2022-08-13

**Authors:** Wenlin Wang, Weiguang Long, Yang Liu, Bin Cai

**Affiliations:** Department of Chest Wall Surgery, Guangdong Second Provincial General Hospital, Guangzhou, China; Department of Chest Wall Surgery, Guangdong Second Provincial General Hospital, Guangzhou, China; Department of Chest Wall Surgery, Guangdong Second Provincial General Hospital, Guangzhou, China; Department of Chest Wall Surgery, Guangdong Second Provincial General Hospital, Guangzhou, China

## Abstract

Several operations for asphyxiating thoracic dystrophy (ATD) have been used previously, but they all have disadvantages. We report a 25-year-old male ATD patient who had significant depressions on both sides of thorax. We designed a special operation for him, which had not only eliminated the depression, but also increased the volume of the thorax. The results show that this operation is a reasonable choice for this kinds of patient.

## INTRODUCTION

Asphyxiating thoracic dystrophy (ATD) is a rare autosomal recessive disease reported by Jeune in 1955 [1]. It’s narrow thorax is the main factor threatening the life of patients, and operation is recognized as the only effective method of treatment [2, 3]. Several operations have been used in clinic in the past [1–4]. However, they all have disadvantages. Here we report a 25-year-old male ATD patient with severe depressions on both sides of thorax. We designed a unique operation for him and achieved satisfactory results.

## CASE REPORT

**Figure 1:**
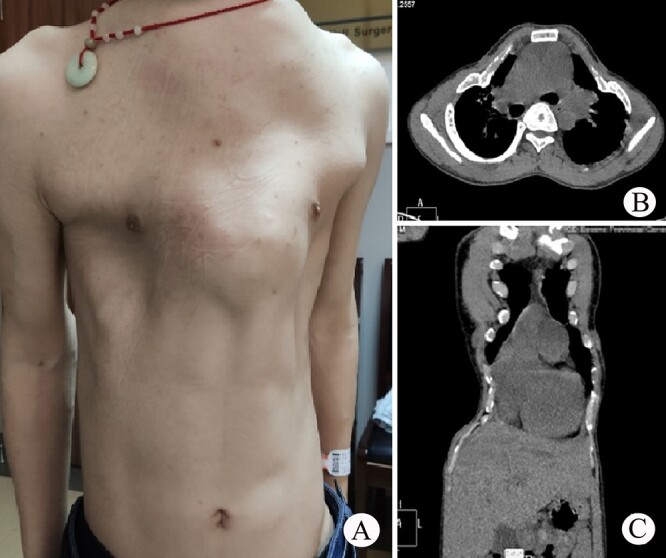
(**A**) Thorax appearance before operation; and (**B**) and (**C**) CT scan of thorax.

**Figure 2:**
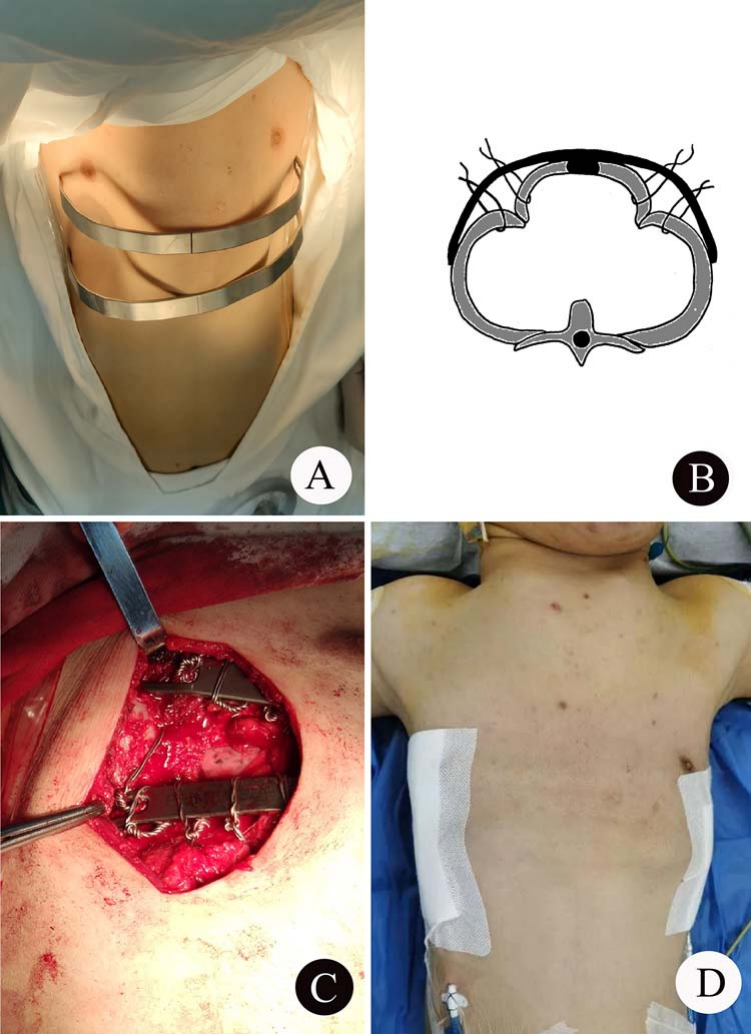
(**A**) The shape of the two steel bars and the approximate position; (**B**) schematic diagram of operation; (**C**) the operation picture; and (**D**) thorax appearance after operation.

**Figure 3:**
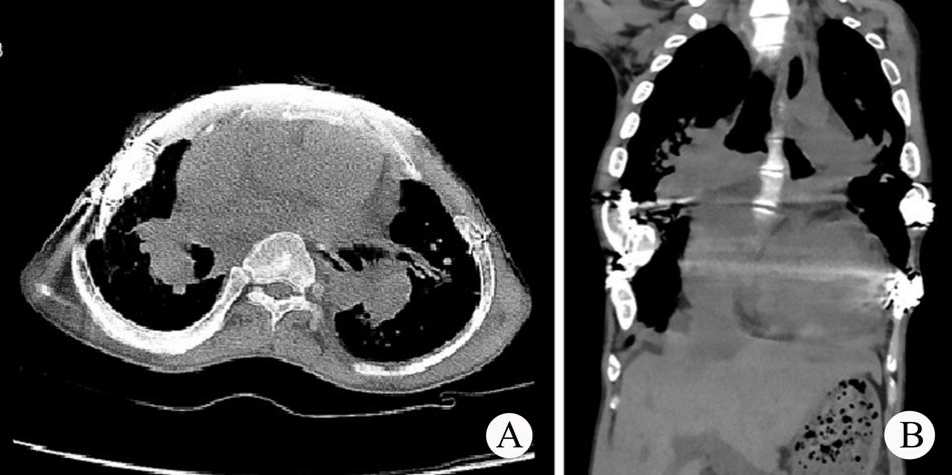
(**A**) and (**B**) Postoperative CT scan of thorax.

The patient was a 25-year-old man who had respiratory discomfort after birth and was diagnosed as ATD at that time. His respiratory symptoms were obvious in childhood. Hypoxia always appeared when crying but disappeared after rest. After age of 5, his condition gradually stabilized. However, after puberty, his respiratory symptoms reappeared and worsened 1 year before admission. He was admitted to our hospital for surgery finally. Physical examination showed that his thorax was narrow and small (Fig. 1A), and his height was 145 cm and chest circumference was only 63 cm. Imaging examination revealed that there were obvious depressions on both sides of the chest wall (Fig. 1B and C). The operation was carried out under general anesthesia, and performed on both sides of chest wall simultaneously. Two longitudinal incisions were performed on each sides of the chest wall respectively in the axillary midline to expose the ribs and costal cartilages. Two tunnels were made in front of the sternum but beneath the soft tissues of the anterior chest wall, and two arc-shaped steel bars were put into the tunnels, respectively. Both sides of the bars were located in front of the depressions. The structures in the depressions were lifted and fixed on the bars (Fig. 2A–C), and the depressions disappeared completely. After the incisions were closed, the operation was completed (Fig. 1D). The symptoms improved significantly, the blood oxygen saturation maintained over 92%, the chest circumference increased to 70 cm and the appearance of the chest was basically normal postoperatively. The patient was discharged 25 days after operation. Followed up for 1 year, there were no symptoms during daily activities, but there were hypoxia after vigorous activities. Imaging examination showed that the shape of the thorax was significantly improved (Fig. 3A and B).

## DISCUSSION

The main damage of ATD comes from the restriction of two lungs. Therefore, the purpose of operation is to relieve the restriction of lung tissue. Generally, the previous surgical methods can be divided into three categories: median thoracic expansion (MTE) [2], lateral thoracic expansion (LTE) [3] and orthopedic surgery [4]. MTE is to split the sternum from the middle and then support it with different materials to increase the thorax volume [2]. LTE is to divide the ribs in a staggered fashion and then made the adjacent long ends of the ribs opposed and secured with titanium bar, which can also increase the thorax volume [3]. Orthopedic surgery is directly aimed at the depression on the chest wall, which can directly increase the volume of the thorax by eliminating the depression [4]. Although all these operations have definite effects, they also have some disadvantages.

By April 2022, we had performed surgical treatment on 32 patients with ATD. After analyzing the chest wall structure of these patients, we found that ATD can be divided into two types: type I, there is no obvious depression on the lateral chest wall; type II, there is obvious depression on the lateral chest wall. MTE and LTE cannot eliminate lateral chest wall depression, so they can only be used for type I ATD. The orthopedic surgery is specially used to correct the depression on the chest wall; therefore, it is suitable for type II ATD. Our patient is a typical type II ATD, so we designed a new orthopedic operation for him. We directly use steel bars to suspend the depressions. When the depressions are eliminated, the circumference of the thorax increases, the volume of the thorax expands and the restriction of the lung tissue is relieved. Our results show that this operation is a relatively safe, simple and effective choice. However, due to the limited clinical experience, its actual effect still needs to be further proved.

## CONFLICT OF INTEREST STATEMENT

None declared.
